# Potential for Lung Recruitment Maneuvers Estimated by the Cytokines in Bronchoalveolar Lavage Fluid in Acute Respiratory Distress Syndrome

**DOI:** 10.1155/emmi/5442038

**Published:** 2025-02-10

**Authors:** Minjin Shen, Jiaping Huai

**Affiliations:** ^1^Department of Gastroenterology, Affiliated Jinhua Hospital, Zhejiang University School of Medicine, Jinhua 321000, Zhejiang, China; ^2^Department of Critical Care, Affiliated Jinhua Hospital, Zhejiang University School of Medicine, Jinhua 321000, Zhejiang, China

**Keywords:** ARDS, bronchoalveolar lavage fluid, cytokines, recruitment maneuvers

## Abstract

**Objective:** Lung recruitment maneuvers (RMs) is an important treatment for acute respiratory distress syndrome (ARDS) patients; however, assessing lung recruitability is imperative to avoid biotrauma and hemodynamic instability. This study aims to investigate whether the cytokine levels in the bronchoalveolar lavage fluid (BALF) of ARDS patients can serve as an indicator of their lung recruitability.

**Methods:** This study included ARDS patients who received mechanical ventilation for over 24 h. Patients were categorized into lung recruitment maneuver effective (RM-E) group and lung recruitment maneuver noneffective (RM-N) group. Interleukin-6 (IL-6), interleukin-8 (IL-8) and interleukin-10 (IL-10) in BALF, lung ultrasound (LUS) scores, and the oxygenation index (P/F) were measured. The differences in cytokine levels between the two groups were compared, and correlations between changes in cytokine levels (ΔIL-6, ΔIL-8, and ΔIL-10), ΔLUS, and ΔP/F were analyzed.

**Results:** Sixty-two patients were included in this study (38 in the RM-E group and 24 in the RM-N group). After the RM, compared with the RM-N group, an increase was observed in ΔIL-6 (*p*=0.013), ΔIL-8 (*p*=0.045), ΔIL-10 (*p*=0.039), and ΔLUS (*p*=0.045) in the RM-E group. A positive linear correlation was found between ΔIL-6 and ΔLUS (*r* = 0.504, *p* < 0.001). The area under the lung recruitment potential curve (AUC) predicted by ΔIL-6 was 0.794, the sensitivity was 94.7%, and the specificity was 70.8%. A positive linear correlation was found between ΔIL-6 and ΔLUS (*r* = 0.504, *p* < 0.001). The lung recruitment potential curve's AUC predicted by ΔIL-6 was 0.794, with a sensitivity of 94.7% and specificity of 70.8%.

**Conclusion:** Lower levels of cytokines in BALF were observed in the RM-E group. It is possible that the cytokines in BALF, especially IL-6, could be used to determine the need for RM on the basis of lung recruitability.

## 1. Introduction

The Berlin definition highlights the heterogeneity of acute respiratory distress syndrome (ARDS), characterized by different etiologies, inflammation activation, and lung recruitability [1, 2]. ARDS is a life-threatening condition with significant mortality and morbidity rates [3]. Research has demonstrated that lung recruitment maneuvers (RMs) are more likely to produce favorable outcomes in the early stages of ARDS [4]. However, it is challenging to predict an individual patient's responsiveness to RM, and there are no reliable and easily accessible clinical methods to assess lung recruitability at the bedside. Computed tomography (CT) scans offer morphological information and enable the differentiation between recruitment and inflation, as well as the evaluation of the effect of RM [5]. CT has limitations in clinical practice due to safety concerns such as radiation and transportation. Lung ultrasonography (LUC) is a noninvasive technique that is readily available and simple to use at the bedside, with high specificity and sensitivity to detect lung collapse [6]. Furthermore, it allows for regional analysis of lung RM in both dependent and nondependent regions. Thus, this study employed LUC to evaluate the effect of RM.

Previous studies have indicated that RM can help alleviate hypoxemia by increasing the end-expiratory lung volume (EELV) [3]. However, this technique may also cause barotrauma or volutrauma [7]. Both animal and clinical studies have found that high airway pressure during RM is linked to the release of inflammatory cytokines [8, 9]. This is likely due to lung over-distension caused by the high airway pressures, which can cause the redistribution of pulmonary blood flow toward atelectatic regions and induce an inflammatory response [10].

While RM is a common intervention for ARDS, there is a scarcity of studies exploring the relationship between inflammatory cytokines in BALF and RM. This study aims to investigate the immunological effects of lung RM by monitoring changes in inflammatory cytokines in BALF.

## 2. Methods

### 2.1. Study and Patients

This prospective, single-center, observational study was conducted in the medical ICU at the Affiliated Jinhua Hospital, Zhejiang University School of Medicine, China. The study received approval from the hospital's local ethics committee (2019–022-001), and informed consent was obtained from each patient or their legal substitute decision maker before any study procedure was initiated.

ARDS patients who met the Berlin definition criteria were recruited for this study between March 1, 2020, and April 7, 2022 [1]. Inclusion criteria were (1) age > 18 and < 85 years; (2) presence of moderate or severe ARDS (PaO_2_/FiO_2_ ≤ 200 mmHg); and (3) mechanical ventilation for more than 24 h. Exclusion criteria were (1) age < 18 or > 85 years; (2) undrained pneumothorax; (3) hemodynamic instability (norepinephrine or epinephrine > 1 μg/kg/min); (4) PaO_2_/FiO_2_ < 80 mmHg on 100% FiO_2_; (5) severe chronic respiratory disease requiring long-term oxygen therapy or long-term mechanical ventilation; and (6) increased intracranial pressure (> 20 mmHg). Patients were categorized into two groups based on the LUS score after RM: the RM effective group (RM-E group) and the RM noneffective group (RM-N group) [11].

### 2.2. Recruitment Methodology and Assessment

Our study employed the use of low tidal volume ventilation of 6 mL/kg of predicted body weight (PBW) for managing ARDS [12]. Prior to the lung RM, patients were ventilated in the supine position with adequate sedation using a Siemens Servo 300 ventilator. During RM, the mean airway pressure was elevated to 40 cm H_2_O and maintained for 30 s before returning to the previous ventilation strategy [13].

The LUS was performed using an M-Turbo ultrasound machine with a 2- to 5 MHz curved array probe (FUJIFILM SonoSite, Bothell, WA, USA) by two ICU expert physicians who were certified for critical LUS. The examination included both the upper and lower intercostal spaces of the anterior, lateral, and posterior regions of both the left and right chest walls and was conducted anonymously and blindly [14]. The worst ultrasound abnormality detected was considered a characteristic of the area examined. Four ultrasound aeration patterns were defined [15]: (1) normal aeration (N): presence of lung sliding with A lines or fewer than two isolated B lines (0 point); (2) moderate loss of lung aeration: multiple well-defined B lines (B1 lines, one point); (3) severe loss of lung aeration: multiple coalescent B lines (B2 lines, two points); and (4) lung consolidation (C): the presence of a tissue pattern characterized by dynamic air bronchograms (3 points). The LUS score for each region was obtained by averaging the scores of all pertinent intercostal spaces within that region and rounding the result, with a range of 0–3. The global LUS score, ranging from 0 to 36, was derived by adding the scores of all 12 regions [16].

### 2.3. Procedures

Bronchoalveolar lavage (BAL) was performed by passing a fiberoptic bronchoscope through the endotracheal tube after preoxygenating patients with 100% oxygen for 15 min before and during the procedure. In the region of the worst ultrasound score, five separate 30 mL aliquots (totaling 150 mL) of normal saline were instilled and retrieved by hand suction from all participants [17]. Bronchoalveolar lavage fluid (BALF) was collected before and 24 h after RM. After centrifugation at 250*g* for 10 min at 4°C, the BALF supernatant was aliquoted into small volumes and stored at −80°C. The levels of cytokines, including IL-6, IL-8, and IL-10, were measured using enzyme-linked immunosorbent assay (R&D Systems, MN, USA).

### 2.4. Statistical Analysis

Data were presented as either mean ± standard deviation or median values and interquartile ranges. The Mann–Whitney *U* test was used to compare two groups, and the Pearson's correlation test was utilized to determine the *r* correlation coefficient. Discriminatory power of cytokine levels was quantified using the area under the receiver operating characteristic curve (AUROC). Statistical analysis was performed using SPSS 22.0 (SPSS Inc., Chicago, IL, USA), and all tests were 2-sided with *p* values < 0.05 considered statistically significant.

## 3. Results

**Figure 1:**
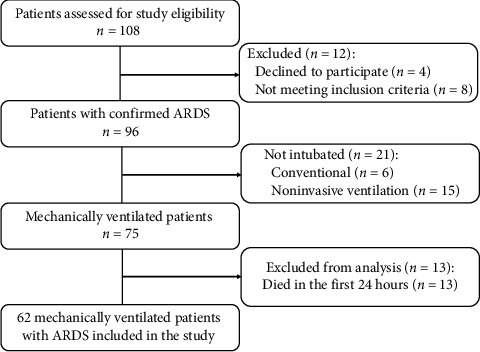
The study cohort selection.

During the study period, 108 patients diagnosed with ARDS were admitted to the ICU, with 62 meeting the study's inclusion and exclusion criteria (Figure 1). Baseline demographic and laboratory characteristics, comorbidities, respiratory parameters, and subgroups of the RM-E and RM-N groups are presented in Table 1. The mean age of the 62 patients (39 males and 23 females) was 65.2 ± 10.34 years, and there were no significant differences in APACHE II Score, respiratory parameters, LUS score, and cytokine levels between the two groups.

IL-6, IL-8, and IL-10 levels in BALF were significantly reduced 24 h after RM compared to pre-RM levels in both the RM-E and RM-N groups (*p* < 0.05, Table 2). LUS score decreased significantly after RM in the RM-E group (*p*=0.008), while no significant difference was observed in the RM-N group (*p*=0.927). Oxygenation improved in both groups, with an increase in the PaO_2_/FiO_2_ ratio from the baseline to post-RM (RM-E: 120.1 ± 24.3–189.1 ± 35.8; *p*=0.04; RM-N: 118.8 ± 22.1–208.9 ± 32.4; *p*=0.029).

Table 3 shows that the variation of IL-6, IL-8, and IL-10 (ΔIL-6, ΔIL-8, and ΔIL-10) in BALF after RM was significantly higher in the RM-E group compared with the RM-N group (*p* < 0.05). Similarly, patients with effective RM had a significantly higher ultrasound score variation (ΔLUS) than those with ineffective RM (*p* < 0.05). However, there was no significant difference between the two groups in terms of P/F variation (ΔP/F) (*p*=0.089).

**Figure 2:**
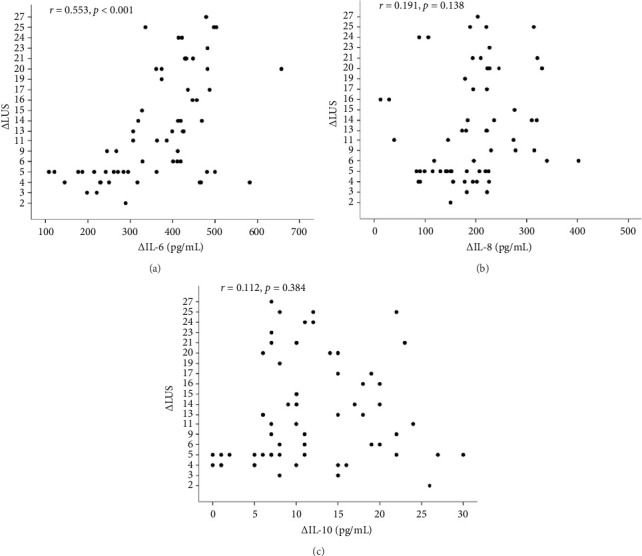
(a) Correlations between ΔIL-6 and ΔLUS score. (b) Correlations between ΔIL-8 and ΔLUS score. (c) Correlations between ΔIL-10 and ΔLUS score. Each closed circle represents an individual patient.

In correlation analyses between ΔIL-6, ΔIL-8, ΔIL-10, and ΔLUS in patients with ARDS, only the ΔIL-6 level in BALF was positively and significantly correlated with ΔLUS (*r* = 0.504, *p* < 0.001, Figure 2(a)). However, ΔIL-8 and ΔIL-10 were not statistically associated with ΔLUS (*r* = 0.023, *p*=0.856 for ΔIL-8, Figure 2(b); *r* = 0.028, *p*=0.827 for ΔIL-10, Figure 2(c)).

Correlation analyses between ΔIL-6, ΔIL-8, ΔIL-10, and ΔLUS in patients with ARDS revealed that only ΔIL-6 level in BALF was positively and significantly correlated with ΔLUS (*r* = 0.504, *p* < 0.001, Figure 2(a)). However, ΔIL-8 and ΔIL-10 were not statistically associated with ΔLUS (*r* = 0.023, *p*=0.856 for ΔIL-8, Figure 2(b); *r* = 0.028, *p*=0.827 for ΔIL-10, Figure 2(c)).

**Figure 3:**
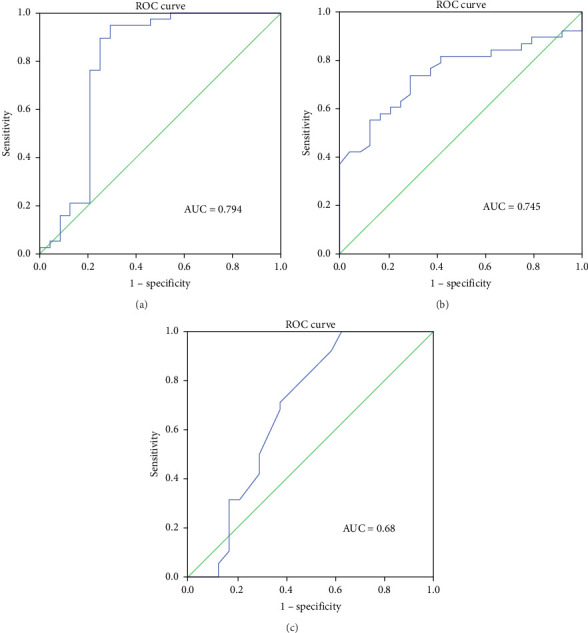
(a) Receiver operating characteristic curve (ROC) predicting the RM effective potential with the ΔIL-6. (b) ROC predicting the RM effective potential with the ΔIL-8. (c) ROC predicting the RM effective potential with the ΔIL-10. AUC = area under curve.

ROC curve analysis was performed to determine the predictive ability of ΔIL-6, ΔIL-8, and ΔIL-10 levels in BALF for RM effectiveness. The analysis revealed that the ΔIL-6 level had the highest area under the curve (AUC) value of 0.794 (95% confidence interval [CI] 0.652–0.936, *p* < 0.001, Figure 3(a)), followed by ΔIL-8 with an AUC of 0.745 (95% CI 0.623–0.867, *p* < 0.005, Figure 3(b)) and ΔIL-10 with an AUC of 0.68 (95% CI 0.526–0.835, *p* < 0.05, Figure 3(c)). Using ΔIL-6 to predict RM effectiveness produced sensitivity and specificity values of 94.7% and 70.8%, respectively, with a cutoff value of 301. For ΔIL-8, the sensitivity and specificity were 73.7% and 70.8%, while for ΔIL-10, they were 92.1% and 41.7%.

## 4. Discussion

This study introduces a new bedside method that has the potential to identify ARDS patients who are most likely to benefit from RM. The key findings of this study are (1) detectable levels of IL-6, IL-8, and IL-10 in BALF were decreased after RM and (2) the variation of IL-6 was found to be statistically associated with LUS score variations, which can be used to assess the effectiveness of RM [18].

The multifaceted nature of the impact of mechanical ventilation on pro- and anti-inflammatory mediators necessitates careful consideration of the underlying mechanisms [19]. Injurious mechanical ventilation in ARDS patients can affect both pro- and anti-inflammatory cytokines, leading to simultaneous changes in systemic cytokine levels [20]. The development of the inflammatory response in ARDS is influenced by the balance between pro- and anti-inflammatory factors. Studies have consistently reported elevations in IL-6, IL-8, and IL-10 levels in ARDS patients over the course of the disease [21, 22]. While RM can effectively reverse alveolar collapse and improve gas exchange [23], it can also cause stretch-induced injury and the release of inflammatory mediators, potentially leading to ventilator-associated lung injury [24, 25]. Previous studies have reported that RM may decrease serum levels of IL-6 and IL-10, albeit not significantly [26], while another study found a significant reduction in endotracheal IL-6 levels, suggesting a decrease in lung inflammation [27]. These findings are in agreement with previous results, which demonstrated that combining protective ventilation with lung RM leads to a significant decrease in serum levels of IL-8 and IL-10 after cardiopulmonary bypass in patients undergoing cardiac surgery [28]. While the clinical significance of IL-6, IL-8, and IL-10 levels in BALF during RM in ARDS patients remains unclear, our study revealed a marked decrease in the levels of these cytokines in both the RM-E and RM-N groups. These results suggest that RM may improve the systemic inflammatory response. Although the improvement in oxygenation after RM was not clinically significant in most patients, this finding is consistent with previous studies that have reported varying effects of RM on gas exchange depending on the underlying factors [29].

Although lung RM can improve ventilation and gas exchange in ARDS patients, it can also generate excessive stress on the lungs [7]. We attempted to use cytokines in BALF as a surrogate method for evaluating lung recruitability and found a significant positive correlation between ΔIL-6 and ΔLUS score but not between ΔIL-8 or ΔIL-10 levels and recruitability. This lack of correlation may be due to differences in ventilatory strategies, ARDS stages, or a small sample size. Our results suggest that IL-6 may be a more effective predictor of RM outcome in ARDS patients, but further research is needed to understand the mechanisms underlying cytokine release in the alveolar environment.

Our study has several limitations. First, as with all ultrasound studies, the accuracy of the findings depends on the expertise of the operator. Second, using the bronchoscopy microsampling method to obtain epithelial lining fluid from small airways may have led to different cytokine levels compared to bronchoalveolar lavage [30], and there is no intervention as a control measure in this process. Third, our evaluation of lung recruitability was restricted to a fixed change in PEEP, and a wider range of PEEP would have been necessary to fully assess the potential for recruitment. Furthermore, the 24-h observation period may not be applicable to longer ventilation periods or the clinical setting. Lastly, the small sample size reduces the statistical power of our results, and larger prospective multicentric studies are required to confirm our findings.

In conclusion, our study has demonstrated that lung RM leads to a significant decrease in inflammatory cytokine expression. We found a clinically useful correlation between lung recruitability and inflammatory markers, particularly in the variation of IL-6 levels, which may serve as a valuable bedside tool. Our findings suggest that measuring the variation in BALF IL-6 levels may be useful in evaluating the efficacy of RM, as we observed a positive correlation between the variation in BALF IL-6 levels and improvement in the LUS score in patients with ARDS.

## Figures and Tables

**Table 1:** Baseline characteristics of the patients.

	Overall (*n* = 62)	RM-E (*n* = 38)	RM-N (*n* = 24)	*p* value
Demographics				
Age, years	65.2 ± 10.4	64.3 ± 10.4	66.5 ± 10.3	0.415
Males	39 (62.9)	25 (65.8)	14 (58.3)	0.561
Comorbidities				
Hypertension	47 (75.8)	29 (76.3)	18 (75)	
Diabetes mellitus	20 (32.3)	13 (34.2)	7 (29.2)	
Congestive heart failure	4 (6.5)	3 (7.9)	1 (4.2)	
COPD	10 (16.1)	7 (18.4)	3 (12.5)	
Cerebrovascular disease	19 (30.7)	11 (29)	8 (33.3)	
History of cancer	2 (3.2)	2 (5.3)	0 (0)	
APACHE II score	20.3 ± 3.4	20.5 ± 3.5	19.9 ± 3.3	0.584
VT (mL)	342.4 ± 53.4	336.6 ± 50.2	351.5 ± 58	0.396
PEEP (cmH_2_O)	11.9 ± 1.5	11.9 ± 1.6	11.9 ± 1.4	0.671
P_plat_ (cmH_2_O)	23.2 ± 3.3	23.6 ± 3.4	22.7 ± 3.3	0.713
FiO_2_ (%)	61.3 ± 7.1	62.9 ± 6.4	58.8 ± 7.1	0.546
P/F (mmHg)	119.6 ± 23.3	120.1 ± 24.3	118.8 ± 22.1	0.366
LUS score	24.1 ± 4.4	24.5 ± 4.6	23.5 ± 4.2	0.502
IL-6 (pg/mL)	698 ± 133.2	711.6 ± 122	676.4 ± 149.5	0.971
IL-8 (pg/mL)	514 ± 94.8	507.1 ± 102.7	524.8 ± 81.6	0.247
IL-10 (pg/mL)	28.6 ± 9.5	27.9 ± 9.5	29.8 ± 9.5	0.766

*Note:* Data are presented as the mean ± standard deviation or *n* (%). P_plat_ = plateau pressure, P/F = oxygenation index.

Abbreviations: APACHE = acute physiologic and chronic health evaluation, COPD = chronic obstructive pulmonary disease, FiO_2_ = fraction of inspired oxygen, IL-6 = interleukin-6, IL-8 = interleukin-8, IL-10 = interleukin-10, LUS = lung ultrasound, PEEP = positive end expiratory pressure; VT = tidal volume.

**Table 2:** Comparison of cytokine levels in pre-RM and 24 h post-RM.

	IL-6	IL-8	IL-10	LUS score	P/F
RM-E (*n* = 38)					
Pre-RM	711.6 ± 122	507.1 ± 102.7	27.9 ± 9.5	24.5 ± 4.6	120.1 ± 24.3
Post-RM	301.5 ± 94.3	291.9 ± 70.3	14.8 ± 5.5	8.5 ± 3.4	189.1 ± 35.8
*p* value	0.047	0.018	<0.001	0.008	0.040
RM-N (*n* = 24)					
Pre-RM	676.4 ± 149.5	524.8 ± 81.6	29.8 ± 9.5	23.5 ± 4.2	118.8 ± 22.1
Post-RM	389.1 ± 68.9	367.4 ± 54.1	20 ± 3.5	19.2 ± 4.2	208.9 ± 32.4
*p* value	0.026	0.008	<0.001	0.927	0.029

*Note:* Data are presented as the mean ± standard deviation.

**Table 3:** Comparison of cytokine levels, LUS, and P/F variation.

	ΔIL-6	ΔIL-8	ΔIL-10	ΔLUS score	ΔP/F
RM-E (*n* = 38)	410 ± 76.2	215.2 ± 87.7	13.1 ± 5.6	16 ± 6.1	69 ± 25.4
RM-N (*n* = 24)	287.3 ± 126.9	157.4 ± 47	9.8 ± 8.9	4.4 ± 0.8	90.1 ± 34.6
*p* value	0.013	0.045	0.039	< 0.001	0.089

*Note:* Data are presented as the mean ± standard deviation. ΔIL-6 = variation of interleukin-6 after RM; ΔIL-8 = variation of interleukin-8 after RM; ΔIL-10 = variation of interleukin-10 after RM; ΔLUS = variation of LUS after RM; ΔP/F = variation of P/F after RM.

## Data Availability

The data that support the findings of this study are available from the corresponding author upon reasonable request.
